# Comparative evaluation of antimicrobial-corticosteroid mixture and intracanal cryotherapy on post-instrumentation pain and microbial reduction: A randomized clinical trial

**DOI:** 10.4317/jced.63139

**Published:** 2025-11-30

**Authors:** Mohammed F. Habib, Maged M. Negm, Alaa A. El-Baz, Sherifa T. Salem

**Affiliations:** 1 PhD candidate, Department of Endodontics, Faculty of Dentistry, Cairo University, Egypt; 2 Professor, Department of Endodontics, Faculty of Dentistry, Cairo University, Egypt; 3 Associate professor, Department of clinical and chemical pathology, Faculty of Medicine, Cairo University, Egypt

## Abstract

**Background:**

The risk of pain and flare up following root canal treatment of asymptomatic necrotic teeth remains an unsolved issue despite the recent advances in canal preparation and obturation procedures. The present study evaluated the role of two final irrigations in minimizing such risk. Aim: To compare the effect of final irrigation with antimicrobial - corticosteroid mixture with that of cryotherapy on post-instrumentation pain and microbial reduction in patients with single canalled teeth with necrotic pulps and asymptomatic apical periodontitis.

**Material and Methods:**

Twenty-eight patients with asymptomatic necrotic single-canalled teeth participated in the study. After diagnosis, access cavity preparation and chemico-mechanical preparation with rotary files and 2.5% NaOCl irrigation, patients were randomly allocated to receive a final irrigation of either a mixture of levofloxacin, dexamethasone and fluconazole or intracanal cryotherapy. Patients recorded their postoperative pain on an 11-point numerical rating scale up to 72 hours. Microbial samples were collected at 3 stages: preoperatively, post-instrumentation and post- final irrigation.

**Results:**

The two groups showed no significant increase in postoperative pain intensity at all time intervals compared to the preoperative pain intensity. The two groups showed mild pain intensity at 6 and 12 hours postoperatively with no significant difference between them. No pain was reported in both groups at 24, 48 and 72 hours postoperatively. The post-final irrigation samples showed significant bacterial and candidal load reduction in both groups.

**Conclusions:**

Final irrigation with antimicrobial-corticosteroid mixture and cryotherapy following 2.5% NaOCl prevented the rise of the postoperative pain intensity and minimized the intracanal microbial loads with no significant differences between both irrigations.

## Introduction

The risk of experiencing pain after root canal treatment might range from 3% to 58% (1). Teeth with necrotic pulps are less common to cause intraoperative pain unless the tooth is associated with symptomatic apical periodontitis. However, the situation becomes more frustrating to the patients and stressful to the dentists when patients present to the clinic without clinical symptoms, and later, suffer from severe postoperative pain or flare up. Among the attempts studied to minimize postoperative pain was the alteration of the concentration of sodium hypochlorite irrigation and the use of alternative irrigating solutions. Sodium hypochlorite remains the most widely used irrigant in root canal treatment owing to its dual benefits of organic tissue dissolution and antimicrobial efficacy. However, its application is associated with several limitations, including significant cytotoxicity, particularly when in contact with periradicular tissues. Despite its broad-spectrum antimicrobial activity, sodium hypochlorite may not completely eliminate all microbial content within the root canal system. Furthermore, sodium hypochlorite had been linked to harmful changes in the structural integrity of dentine such as reduced microhardness and flexural strength resulting from degrading the organic collagen matrix (2). Such changes might increase the susceptibility of root canal treated teeth to microcrack propagation and subsequent fracture under heavy occlusal stresses (2). Antibiotic irrigation represents a valuable adjunct in root canal disinfection, offering targeted microbial control, enhanced biofilm disruption, and improved clinical outcomes, especially in cases where conventional mechanical and chemical debridement methods are insufficient to achieve thorough canal sterilization. Tetracycline was introduced in MTAD by Torabinejad et al., 2003 (3), and its use as a final rinse following 1.3% NaOCl irrigation showed similar postoperative pain reduction as the combination of 5.25% NaOCl and 17% EDTA (4). Unfortunately, MTAD possesses the disadvantage of having short shelf life after mixing the powder with the liquid even if kept in a refrigerator. This drawback in addition to its relatively higher cost compared to other antibiotics limits its use in daily practice. Another triple antibiotic mixture of tetracycline, ornidazole and ciprofloxacin was tried as a final irrigation and showed a bacterial load reduction of only 66% (5). Levofloxacin is a type of quinolones that shows a broader spectrum against gram positive bacteria compared to ciprofloxacin with a lower minimum inhibitory concentration, thus providing greater antibacterial efficacy than ciprofloxacin at a smaller dose (6). This study suggested mixing levofloxacin with fluconazole antifungal agent, and corticosteroids which can successfully reduce the frequency and severity of postoperative pain after endodontic procedures by blocking the inflammatory pathway. Another attempt that had been tried was cryotherapy. Lowering the tooth temperature had a significant effect in reducing postoperative pain in addition to being safe and of low cost (7). Accordingly, this study was conducted to test the null hypothesis that there would be no difference between final irrigation with antimicrobial - corticosteroid mixture and cryotherapy in post-instrumentation pain and microbial reduction.

## Material and Methods

Ethical approval and trial registration: The study was conducted in the outpatient clinic, department of Endodontics, Faculty of dentistry, Cairo University, Egypt, between July-2024 and January-2025. The protocol of this trial followed the Helsinki declaration and was approved by the research ethics committee of the faculty of Dentistry, Cairo University (Approval number: REC 4-5-23) and was registered online on https://clinicaltrials.gov/study/NCT05739682. The trial was reported following the CONSORT statement guidelines. Eligibility Criteria: The inclusion criteria were adult patients within the age range of 18 - 50 years, good physical health corresponding to ASA class I or II, having a single-canalled tooth diagnosed with necrotic pulp and asymptomatic apical periodontitis, ability to rate their pain on the NRS scale and agreement to participate in the trial by signing the informed consent. The exclusion criteria were the presence of intraoral or extraoral swelling, open apex, severe periodontal disease, radiographic evidence of root resorption, canal calcification or a periapical lesion larger than 5mm in diameter. After history taking, diagnosis of necrotic pulp with asymptomatic apical periodontitis was confirmed by cold pulp testing using Endo ice (Maquira Indústria de Produtos Odontológicos S.A, Brazil) showing lack of response, and percussion test showing no pain. Sample size calculation: Based on the study by Vera et al., 2018, the mean and standard deviation values of postoperative pain intensity at 24 hours were 2.02 (1.5) in the cryotherapy group (8). Assuming a minimum clinically significant mean difference of 2 NRS scores, a type I error of 0.05, a study power of 0.8, and a 40% increase to compensate for possible dropouts, a total sample of 28 subjects was required in order to reject the null hypothesis that there would be no difference in pain intensity between the two final irrigations of the present study. Randomization: A random table with two columns was generated online using www.random.org by an endodontist who was not involved in the trial. After the operator enrolled an eligible participant and initiated the root canal treatment up to the final irrigation step, a phone call was then made to the endodontist to determine the allocation of the participant to either study group. Blinding: As the antimicrobial-corticosteroid mixture needed to be freshly prepared, and the cryotherapy could be easily felt by the operator, blinding of the operator was not possible. However, the participants who were also the assessors of the pain outcome were blinded to the allocated interventions. The microbial samples were sent to the lab with just the patient's identification number and either a code "A" or "B" for masking the type of the intervention used. Root canal treatment procedures: The clinical trial procedures, risks and benefits were explained to the eligible participants. They were then requested to sign the informed consent, and their baseline data were then recorded. All root canal treatments were done in two visits by the same endodontic specialist. The affected tooth was anesthetized using 4% articaine with 1: 100 000 epinephrine. The tooth was isolated with rubber dam. Caries was removed completely using sterile burs and excavator. The operating field including the tooth, the clamp and the rubber dam sheet were disinfected with 30% H2O2 followed by 5.25% NaOCl. The access cavity was prepared with other sterile round burs and tapered diamond stones. 1 ml of 0.9% sterile saline was used for canal irrigation to allow for taking the 1st microbial sample. Preoperative microbial sample was collected before the canal preparation using 6 sterile paper points of size #25.02 allowed to reach the apical third and left in the canal for 1 minute. The paper points were then transferred to sterile tubes containing 20ml of brain heart infusion (BHI) broth. The coronal two thirds of the canal were flared using the orifice opener size 25 taper 8% (Hyflex, Coltène/Whaledent GmbH, Germany.). Working length was determined using an electronic apex locator (Woodpex III gold, Guilin Woodpecker Medical Instrument CO.,LTD., China.) with the aid of manual size 15 hand K-file (MANI, INC., Industrial Park, Utsunomiya, Tochigi, Japan.). The canal was prepared with Hyflex rotary files up to size #40.04. The canal was irrigated between successive rotary files with 3 ml of 2.5% sodium hypochlorite delivered through a 30-gauge side vented needle. After complete instrumentation, the canal was irrigated with 3 ml of saline. Post-instrumentation microbial samples were collected from root canals as described earlier. Patients were assigned to either the Mix group or the Cryo group according to the random sequence for final irrigation as follows: Mix Group: A mixture of l ml levofloxacin (Tavanic, Sanofi-Aventis Deutchland GmbH, Germany.), 1 ml of fluconazole (Sunny Fungal, Sunny pharmaceutical, Egypt.) and 1 ml of dexamethasone sodium phosphate (Dexamethasone, Amriya for pharmaceutical industries, Egypt.) was passively delivered into the canal using a 30-gauge side vented needle reaching 1mm short of the working length. The solution was then kept inside the canal for 5 minutes. Cryo Group: Using a 30-gauge side vented needle reaching 1mm short of the working length, each canal received 20 ml of 2.5°c cold saline for 5 minutes (9). Post-final irrigation microbial samples were collected from root canals using 6 sterile paper points as previously described. The canal was then dried with sterile paper points. The access was sealed with a dry cotton pellet and temporary restoration. In the second visit, as patients were confirmed asymptomatic, the affected teeth were anesthetized, isolated and then irrigated with 17% EDTA for 1 min followed by 5.25% NaOCl activated with sonic eddy tip and finally flushed with saline. After clinical and radiographic confirmation of master cone fit, the canals were obturated by lateral compaction technique. Assessment of outcomes: The primary outcome was post-instrumentation pain intensity recorded at 6, 12, 24, 48 and 72 hours. Patients received a pain chart containing 5 numerical rating scales, and were instructed to rate their pain levels at the corresponding time intervals on a scale from 0 to 10. Secondary outcomes included the incidence of post-final irrigation positive bacterial and candidal cultures and the microbial load in CFUs/ml. Cultures from collected paper points were inoculated in sterile brain heart infusion (BHI) broth vials which were dispersed with vortex for 30 seconds. For bacterial load assessment, the BHI broth solution was serially diluted one-tenth dilution, plated onto the 5% sheep blood agar medium and incubated at 37°C aerobically for 24 hours. The resultant bacterial growth was quantified by evaluating the number of colonies, and the number of CFU/ml of each dilution was calculated for each sample (10). For candidal load assessment, the BHI broth solution was serially diluted one-tenth dilution, plated onto Sabouraud dextrose agar medium and incubated at 25°C aerobically. The resultant candidal growth was quantified by evaluating the number of colonies, and the number of CFU/ml of each dilution was then calculated for each sample (11). Statistical analysis: Categorical variables were presented as frequencies and percentages, and continuous data as mean, standard deviation, median and range (minimum - maximum). Between group differences were assessed using Chi square test for categorical data and Mann-Whitney U test for continuous data with the Hodges-Lehmann estimator of the median difference and its 95% CI. Paired comparisons were performed using Wilcoxon signed-rank test. Statistical significance level was set at p &lt; 0.05. Statistical analysis was performed using IBM SPSS Statistics for Windows, version 25.0 (IBM Corp., Armonk, NY, USA).

## Results

**Figure 1:**
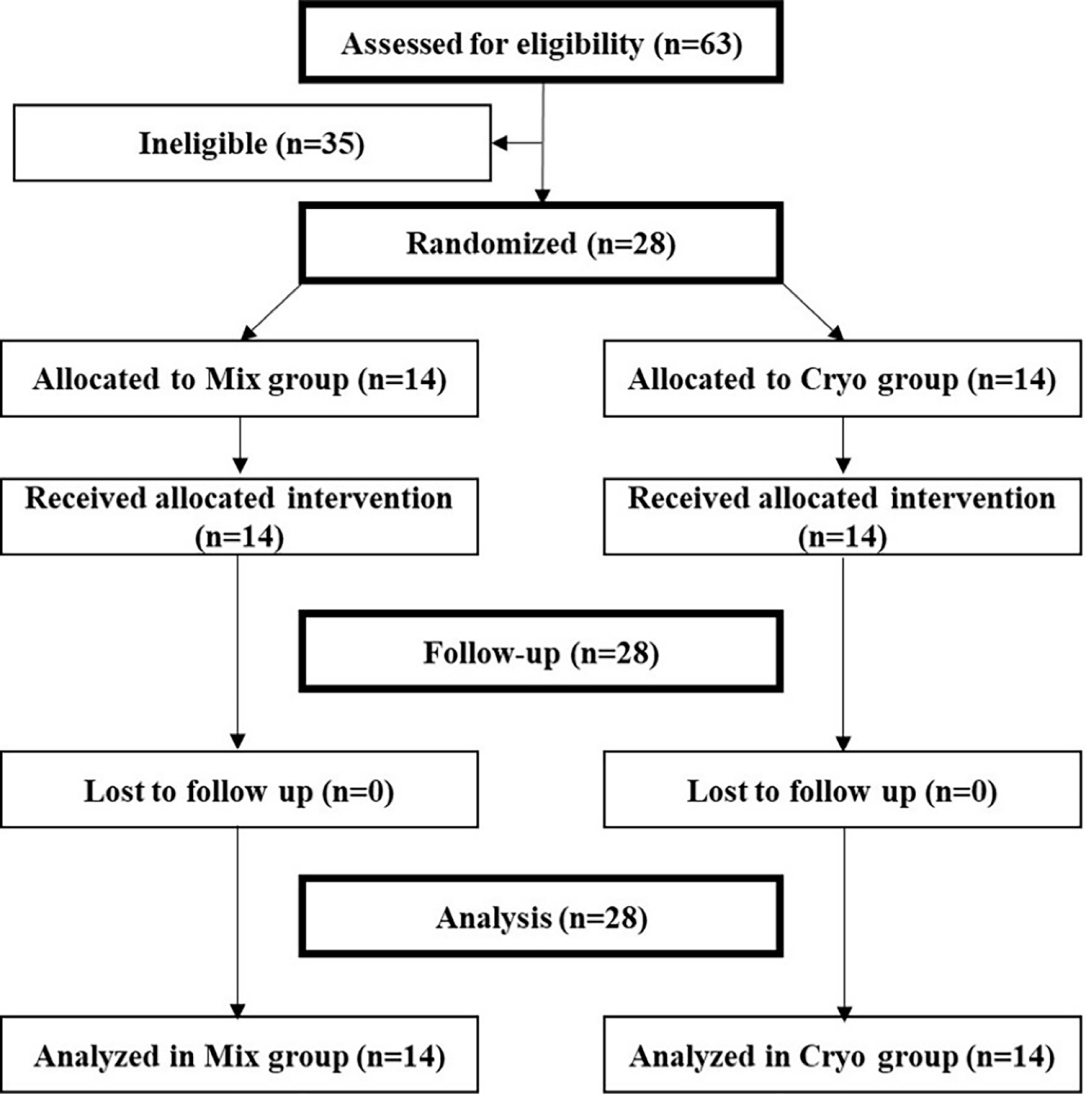
Consort flow diagram.

All 28 participants enrolled in the study were included in the final analysis following the intention-to-treat principle as illustrated in the CONSORT flow diagram in Figure 1.

1

Table 1 shows descriptive statistics of baseline data with no significant differences between the two groups.

Table 1

Table 2 showed the descriptive statistics of post-instrumentation pain intensities at 6, 12, 24, 48 and 72 hours with no significant difference between the two groups.

Table 2

All patients in the two groups reported no pain at 24, 48 and 72 hours. Within the two groups, there were no significant differences between the preoperative pain intensity and post-instrumentation pain intensity at all time intervals. Table 3 shows that there were no significant differences in the incidence of positive bacterial cultures, and the mean bacterial load between the two groups at the post-instrumentation samples. Both groups showed complete absence of positive bacterial cultures at the final irrigation samples.

Table 3

Moreover, the two groups showed absence of positive candidal cultures at the post-instrumentation and post-final irrigation samples.

## Discussion

Postoperative pain has been extensively studied in the literature and its complete elimination has always been a goal for endodontists that has not yet been achieved. Postoperative pain was found to be highly associated with increased prevalence and severity of preoperative pain (12 , 13). In addition, Onay et al., 2015 (14) found that necrotic teeth without periapical pathosis represented high incidence of postoperative flare-ups (6%). Accordingly, only asymptomatic patients were included in this study in order to isolate and evaluate the actual effect of the tested interventions after elimination of preoperative pain as a potential confounder. Up till now, sodium hypochlorite is irreplacable despite its previously mentioned drawbacks especially when used in higher concentrations. Accordingly, the decision in this study was made to use a low concentration of 2.5% NaOCl to minimize the risk of postoperative pain (15). In order to elevate the efficacy of irrigation, the 2.5% NaOCl was followed by one of the two final irrigations tested, either the antibiotic-corticosteroid mixture or the cryotherapy. The word Cryotherapy refers to treatment by cooling. Cryotherapy has more than one application in the field of dentistry. In an in vitro study, Vera et al., 2015 found that 2.5°c cold saline irrigation applied inside the prepared root canal with the microcanula of Endovac device for 5 minutes resulted in reducing the temperature of the apical 4mm of the root surface by 10°c and maintained this reduction in temperature for 4 minutes (16). A systematic review that was conducted by Sadaf et al., 2020 included 8 randomized clinical trials concluded that cryotherapy resulted in significant pain reduction at 6 and 24 hours following root canal treatment (7). However, all included studies were conducted on patients with vital pulps except for the study by Vera et al., 2018 which included necrotic pulps with symptomatic apical periodontitis (8). Two other systematic reviews by Monteiro et al., 2021 and Hespanhol et al., 2022 came to similar conclusions (17 , 18). The cryotherapy technique used in this study was that suggested by Keskin et al., 2017 (9) without the use of Endovac device. The selection of this technique was preferable due to its simpler application without compromising the benefits of pain reduction. The present study further confirmed the effect of cryotherapy on minimizing postoperative pain. Starting with totally asymptomatic patients, cryotherapy succeeded to show no significant increase in postoperative pain. Pain intensity did not exceed mild levels during the initial 12 hours, and by 24 hours patients reported no pain. These results showed resemblance with Keskin et al., 2023, and Iparraguire et al., 2024 where both studies included patients with asymptomatic apical periodontitis and their postoperative pain intensities following intracanal cryotherapy were only limited to less than 1 score following cryotherapy (19 , 20). Moreover, no pain was reported at 48 hours in the study by Keskin et al., 2023 (19) and at 72 hours in the study by Iparraguire et al., 2024 (20). Three possible hypotheses were suggested to explain how cryotherapy can result in such reduction in postoperative pain. First, cold application causes vasoconstriction, which in turn reduces the amount of blood flow and inflammatory exudate in the periapical tissues and thus significantly reducing the postoperative pressure and pain. Second, cryotherapy was found to reduce the excitation threshold of the periapical nociceptors, which reduces the action potentials transmitted to the trigeminal ganglion and reducing the perceived pain. The third possible mechanism is the reduction of cell metabolism, resulting in reducing the cell biochemical reactions and the rate of oxygen consumption which in turn reduces the amount of free radicals produced and thus, minimizing further tissue damage (21). Most reported clinical trials evaluating cryotherapy were concerned with postoperative pain. To our knowledge, the present study is the first to clinically evaluate the antimicrobial effect of cryotherapy showing a significant bacterial load reduction to minimal levels when combined with 2.5% NaOCl. This was in agreement with the findings by Mandras et al., 2023 in vitro study where the combination of cryotherapy with 5% NaOCl showed a significant reduction of E. faecalis bacteria (22). Another adjunctive method used in this study to minimize postoperative pain and enhance the root canal disinfection is the antimicrobial - corticosteroid mixture irrigation. This mixture was composed of levofloxacin antibiotic, fluconazole antifungal and corticosteroid anti-inflammatory agents. Different mixtures of antibiotics and corticosteroids were previously evaluated by Negm, 2001 and Ehrmann et al., 2003 and showed significant reduction in postoperative pain (23 , 24). However, the mixtures were used as intracanal medications, while in the present study the mixtures were used as irrigations to evaluate their effect for future use in single visit root canal treatments. The use of an antibiotic as a final root canal irrigant had been previously introduced as doxycycline in the MTAD solution (3). The benefits of antibiotic irrigation include increasing the spectrum and reducing the required amount or concentration of the NaOCl used. Levofloxacin is a synthetic isomer of the quinolone ofloxacin that was selected in this study as it is a broad-spectrum antibiotic that is effective against gram positive, gram negative, atypical bacteria and bacteria that are resistant to penicillins and macrolides. The drug acts by inhibiting type II topoisomerase enzyme interfering with bacterial critical processes such as DNA transcription, replication, recombination and repair. The combination of levofloxacin and dexamethasone has been previously applied in ophthalmology and was proven effective in controlling inflammation, preventing infection with minimal risk of microbial resistance (25). Moreover, the addition of dexamethasone to fluconazole had demonstrated a synergistic antifungal activity against Candida albicans (26). However, as no previous investigations evaluated the clinical use of the 3-drug mixture as root canal irrigation, thus, the solution was immediately prepared before application to ensure its stability and effectiveness. The antimicrobial results of the Mix group in this study showed a significant bacterial load reduction when used as adjunctive irrigation following 2.5% NaOCl. This result showed similar effect to Sameh et al., 2023 where 99.3% E. Faecalis bacterial load reduction was detected in vitro following the use of a new triple antibiotic mixture composed of levofloxacin, tinidazole and tigycycline (27). Moreover, the results of the present study showed superior bacterial load reduction compared to the study by Jain et al., 2015 which showed a 66% bacterial load reduction achieved by the ordinary antibiotic irrigation mixture of ciprofloxacin, ornidazole and tetracycline used in patients with non-vital single rooted asymptomatic teeth (5). In addition, the mixture contained dexamethasone as a corticosteroid in order to reduce postoperative pain. The results of the present study regarding postoperative pain intensity following the use of corticosteroids were in agreement with Solomon et al., 2024 (28), where both studies showed only mild pain at 6 and 12 postoperatively. At 24 hours pain intensity remained mild in the study carried out by Solomon et al., 2024 (28), while in the present study pain completely disappeared by 24 hours postoperatively. This can be explained by including symptomatic patients in the study by Solomon et al., 2024 (28), compared to the only asymptomatic patients included in the present study. In the present study there was no significant difference in pain intensities between the Mix group and the Cryo group at 6 and 12 hours postoperatively, and in both groups, no pain was reported at 24 hours, while in the study by Solomon et al., 2024 the corticosteroid group showed significantly lower pain intensity at 12 and 24 hours than the cryotherapy group (28). Fluconazole was added to the irrigation mixture for its antifungal action. The presence of fungi was significantly associated with root canal infections with Candida albicans being the most abundant species (29). The present study showed that the 2.5% NaOCl irrigation significantly reduced Candida albicans in the root canals so that they could not be detected by the subsequent culture. This is in agreement with Fidalgo et al., 2010 who reported that 5.25% and 2.5% NaOCl showed the highest efficacy against candida albicans compared to the lower concentrations of 1% and 0.5% (30). A potential limitation of this study was its relatively small sample size which might have limited the detection of a significant difference between the study groups. Furthermore, the lack of a control group with no final irrigation could be considered another limitation, however this choice was made as this scenario has been well assessed earlier in the literature. Future studies can be conducted utilizing molecular microbiological methods and to address healing at longer follow-up periods.

## Conclusions

Within the limitations of this study, it can be concluded that both anti-microbial corticosteroid mixture and cryotherapy can limit postoperative pain to mild intensity at 6 and 12 hours and can significantly alleviate pain by 24 hours when used as a final irrigation following 2.5% NaOCl. Both final irrigations can achieve minimal bacterial and candidal growth levels within infected root canals.

## Figures and Tables

**Table 1:** Descriptive statistics and the results of comparisons of baseline data between the two groups.

		Mix group	Cryo group	p-value
Age	Mean (SD)	33.7 (6.3)	36.4 (5)	0.227
	Median (Range)	33 (26 - 45)	37 (27 - 43)	
Gender	Males N (%)	3 (21.4%)	2 (14.3%)	0.622
	Females N (%)	11 (78.6%)	12 (85.7%)	
Tooth type	Anteriors N (%)	8 (57.1%)	6 (42.9%)	0.45
	Premolars N (%)	6 (42.9%)	8 (57.1%)	
Preoperative pain intensity	Mean (SD)	0 (0)	0 (0)	1.0
	Median (Range)	0 (0 - 0)	0 (0 - 0)	
Preoperative bacterial culture	+ve N (%)	14 (100%)	14 (100%)	NA
	-ve N (%)	0 (0%)	0 (0%)	
Preoperative bacterial load (*103 CFUs/ml)	Mean (SD)	64.3 (33.2)	80.7 (26.2)	0.164
	Median (Range)	75 (10 - 100)	90 (10 - 100)	
Preoperative candidal culture	+ve N (%)	2 (14.3%)	4 (28.6%)	0.357
	-ve N (%)	12 (85.7%)	10 (71.4%)	

SD: standard deviation, Range: (minimum – maximum), N: count of participants, %: percentage of participants within each group, NA: Not applicable.

**Table 2:** Descriptive statistics and the results of Mann-Whitney U test for comparison of postoperative pain intensity at different time intervals between the two groups and Wilcoxon signed rank test for comparison of postoperative pain intensity to preoperative pain intensity within each group.

		Mix group	Cryo group	Median difference(HL, 95%CI)	p-value
Mann - Whitney U test between the two groups					
6 Hours	Mean (SD)	0.9 (1.2)	0.4 (0.6)		0.329
	Median (Range)	0 (0 - 3)	0 (0 - 2)	0 (0,1)	
12 Hours	Mean (SD)	0.6 (1)	0.2 (0.4)		0.401
	Median (Range)	0 (0 - 3)	0 (0 - 1)	0 (0,0)	
24 Hours	Mean (SD)	0 (0)	0 (0)		1.0
	Median (Range)	0 (0 - 0)	0 (0 - 0)	0 (0,0)	
48 Hours	Mean (SD)	0 (0)	0 (0)		1.0
	Median (Range)	0 (0 - 0)	0 (0 - 0)	0 (0,0)	
72 Hours	Mean (SD)	0 (0)	0 (0)		1.0
	Median (Range)	0 (0 - 0)	0 (0 - 0)	0 (0,0)	
Wilcoxon signed rank test within each group					
		Mix Group	Cryo Group		
		p-value	p-value		
Preoperative -	6 Hours	0.066	0.059		
Preoperative -	12 Hours	0.102	0.083		
Preoperative -	24 Hours	1.0	1.0		
Preoperative -	48 Hours	1.0	1.0		
Preoperative -	72 Hours	1.0	1.0		

SD: standard deviation, Range: (minimum – maximum).

**Table 3:** Descriptive statistics and the results of chi square test for comparison of incidence of bacterial and candidal culture and Mann – Whitney U test for comparison of bacterial load between the two groups.

			Mix Group	Cryo Group	p-value
Post-instrumentation	Bacterialculture	+ve N(%)	8 (57.1%)	6 (42.9%)	0.45
		-ve N(%)	6 (42.9%)	8 (57.1%)	
	BacterialLoad (*103 CFUs/ml)	Mean (SD)	2 (2.9)	5.6 (9.9)	0.946
		Median (Range)	1 (0 - 10)	0 (0 - 30)	
	Candidal culture	+ve N(%)	0 (%)	0 (%)	1.0
		-ve N(%)	14 (100%)	14 (100%)	
Post-final irrigation	Bacterial culture	+ve N(%)	0 (%)	0 (%)	1.0
		-ve N(%)	14 (100%)	14 (100%)	
	Bacterial load	Mean (SD)	0 (0)	0 (0)	1.0
		Median (Range)	0 (0 - 0)	0 (0 - 0)	
	Candidal culture	+ve N(%)	0 (%)	0 (%)	1.0
		-ve N(%)	14 (100%)	14 (100%)	

SD: standard deviation, N: count of participants, %: percentage of participants within each group.

## Data Availability

The datasets used and/or analyzed during the current study are available from the corresponding author.
